# Using Chemical Composition and Antioxidant Activity in Evaluation of Enological By-Products According to Type, Vinification Style, Season, and Grape Variety

**DOI:** 10.3390/foods14142405

**Published:** 2025-07-08

**Authors:** Ana Belén Mora-Garrido, María José Jara-Palacios, M. Luisa Escudero-Gilete, María Jesús Cejudo-Bastante

**Affiliations:** 1 Food Colour & Quality Laboratory, Facultad de Farmacia, Universidad de Sevilla, 41012 Sevilla, Spain; amgarrido@us.es (A.B.M.-G.); mjcejudo@us.es (M.J.C.-B.); 2 Department of Analytical Chemistry, Facultad de Farmacia, Universidad de Sevilla, 41012 Sevilla, Spain; mjara@us.es

**Keywords:** grape pomace, lees, grape seed meal, cyclic voltammetry, polyphenols, by-products

## Abstract

Large quantities of oenological by-products, rich in potentially extracted antioxidant compounds, are generated annually in the winemaking industry. With the purpose of their revalorization, different types of by-products (grape pomace, lees, and grape seed meal) from the winemaking industry from three vinification typologies (red, rosè, and white) and four varieties (Tempranillo, Syrah, Airén, and Zalema) in two grape growing seasons (2022 and 2023) were considered. Attention was focused on the content of protein, individual phenolic compounds (anthocyanins, flavonols, hydroxycinnamic acid derivatives, hydroxybenzoic acids, monomeric flavan-3-ols, and procyanidins), and antioxidant activity (DPPH, ABTS, and cyclic voltammetry). The data obtained showed considerable amounts of protein (around 30%) in red lees and a high concentration of phenolic compounds in the by-products, especially anthocyanins and flavonols in the by-products derived from rosè vinifications and flavan-3-ols, procyanidins, and hydroxybenzoic acids in red grape by-products. The antioxidant activity was different between the by-products. Specifically, the electrochemical behavior evaluated by cyclic voltammetry showed some significant differences. Finally, a linear discriminant analysis based on chemical and antioxidant data allowed for differentiating the samples depending on the type of by-product, type of vinification, and variety.

## 1. Introduction

Typical wastes and by-products from wineries include grape pomace (skins and seeds, representing on average about 60% of the total winemaking by-products and stems about 14%), grape solids and fermentation (yeast) lees (25%), wastewaters rich in organic compounds (up to about 15 L/L of wine) [1], carbon dioxide from the fermentation process, exhausted filtration materials and fining agents. Derived from wine industries, approximately seven million tons of grape pomace and lees are created annually worldwide [2]. It is estimated that, from 100 kg of grapes, 20–25 kg of by-products are obtained and that the annual quantity produced in Spain is approximately 1200 million kg (assuming an annual grape production of 6500 million kg, of which is approximately 6000 million).

In recent years, there has been a growing interest in reusing these food wastes not only to reduce their environmental impact through the circular production of by-products but also to entail an economic benefit derived from the reuse of products with added value [3]. Given a circular economy approach, some of these wastes can be successfully “recycled”, reused, or recovered, improving both the economic and environmental conditions. These by-products are typically used for animal feeding, composting, industrial biomass, or distillate production [4]. However, grape pomace and lees are high added-value by-products due to their wide variety of compounds. Grape pomace is composed mainly of fiber, proteins, cellulose, minerals and phenolic compounds. Wine lees are the least studied and exploited waste from the wine industry and are a combination of yeasts, organic acids, insoluble carbohydrates, inorganic salts, lignin, proteins, phenolic compounds, and ethanol [5]. These compounds are susceptible to extraction or transformation and exploitation, with a consequent economic benefit. In addition, this fact could contribute to reducing the environmental impact due to large quantities of grape pomace ending up in landfills, thus generating environmental problems [6].

The phenolic composition of winemaking grapes is influenced by various agroclimatic factors. Several studies highlight how grape variety, agricultural techniques, climatic conditions, and soil type affect the chemical composition of the grapes. Therefore, the phenolic composition of the oenological by-products resulting from winemaking (pomaces and lees) also depends on the starting grapes and the winemaking technique [5,7,8,9].

Oenological by-products have, therefore, proven to be an important source of compounds that possess antioxidant activity and provide beneficial effects on human health [10]. Antioxidant activity has been extensively measured by several in vitro methods, such as spectrophotometric and electroanalytical methods. Among the most-used spectrophotometric methods are ABTS, DPPH, FRAP, and ORAC [11]. These methods consider a single mechanism of action [12,13] which does not adequately inform about the total antioxidant capacity of the compound and, furthermore, does not always correlate with its in vivo activity [14,15].

Among the electroanalytical methods voltammetric techniques, such as cyclic voltammetry (CV), have been successfully applied to measure total antioxidant capacity and have become an alternative to traditional spectrophotometric techniques [16,17]. An electrochemical analysis by voltammetry results in a voltammogram, from which relevant information is obtained in oxidation studies. The potential (E_p_), intensity (I_p_), and peak area (Q) values provide qualitative and quantitative information about the system. Thus, Ep values depend on the nature of the analyte, while I_p_ and Q values are a measure of its concentration [18].

The antioxidant activity of foods rich in antioxidant compounds, such as milk, honey, grape, kiwi, tea, and coffee has been evaluated by cyclic voltammetry [19,20,21,22,23,24]. Voltammetric studies applied to winemaking by-products are scarce. The work carried out by Jara-Palacios et al. [25,26,27] on the antioxidant potential of wines, white grape pomaces, seeds, skins, and stems stands out. In these studies, cyclic voltammetry has been applied to evaluate the antioxidant potential of different varieties of white grape pomace, allowing for the differences between them to be established. Also, it has also been used to evaluate the effect of the solvent on the phenolic extraction from four types of winemaking by-products and to evaluate the influence of adding seeds from grape pomace during winemaking on antioxidant potential of the elaborated wines. Voltammetric techniques have also been used to classify food samples according to the concentration of phenolic compounds [21], having been applied in characterization studies [28], and to monitor the evolution and estimate the consumption of oxidizable species during the aging stage in wines [29,30]. The electrochemical behavior of phenolic acids and flavonoids in aqueous solutions has been evaluated [28,31,32], as well as mixtures of phenolic compounds and amino acids to assess their antioxidant activity [33]. Voltammetry has also been applied to the determination of electroactive biomolecules, such as dopamine, uric acid, and ascorbic acid [34], to study the behavior of some biomolecules in in vitro assays in rat brains [35,36] and to assess the electrochemical behavior of redox enzymes [37]. Therefore, voltammetric techniques are increasingly being used to replace spectrophotometric techniques, and it is important to highlight the usefulness of these electrochemical methods for assessing the integral antioxidant capacity of the sample. As far as we know, no previous studies regarding the antioxidant potential, by cyclic voltammetry, of lees and grape seed meal from winemaking, nor on their comparison with grape pomace, have been published in this respect.

The aim of this work was to evaluate the phenolic composition and antioxidant potential using spectrophotometry and cyclic voltammetry of different winemaking by-products, pomaces, meals, and lees, and to compare them based on different factors: type of by-product, type of winemaking, grape variety, and vintage. This work could allow us to establish the optimal by-products based on their remanent compounds and antioxidant activity in order to be further reused by cosmetic and pharmaceutical industries, among others.

## 2. Materials and Methods

### 2.1. Chemicals and Solvents

Folin–Ciocalteau reagent, formic acid, malvidin-3-glucoside, (+)-catechin, gallic acid, protocatechuic, caffeic acid, quercetin, 2,2′-azino-bis (3-ethylbenzothiazoline-6-sulfonic acid) (ABTS), and disodium hydrogen phosphate anhydrous were acquired (Sigma-Aldrich, Madrid, Spain). HPLC-grade acetonitrile was purchased from VWR International Eurolab S. L. (Barcelona, Spain). Potassium persulfate and potassium dihydrogen phosphate was acquired from Merck (Darmstadt, Germany) and 2,2-diphenyl-1- picrylhydrazyl (DPPH) from Alfa Aesar (Ward Hill, MA, USA). Tetramethylchromane-2-carboxylic acid (Trolox) was acquired from Fluka (Madrid, Spain). Sodium carbonate, sodium chloride, methanol, and hydrochloric acid were acquired (Panreac Química S.L.U., Barcelona, Spain).

### 2.2. Samples

The by-products used in this study were provided by Alvinesa Natural Ingredients, S.A (Ciudad Real, Spain), and Bodegas Nuestra Señora del Socorro (Rociana del Condado, Huelva, Spain). The samples corresponded to grape pomace (P), lees (L), and grape seed meal from the grape pomace industry (M) and from the grape varieties Airén (A), Zalema (Z), Tempranillo (T), Syrah (Y), and considering the addition of chips (C). Samples derived from three types of vinification (red, R; rosè, Rs; and white, W) in two grapevine-growing seasons (2022 and 2023) were considered. The samples are summarized in Table 1. All samples were lyophilized and finely milled.

### 2.3. Protein Content

The total nitrogen content of the by-products was determined, in triplicate, using the standard Kjeldahl method [38] in a MicroKjeldahl System (J.P. Selecta, Barcelona, Spain). The factor used for the conversion of total nitrogen content to protein content was 5.75 [39]. The results were expressed as percentages with respect to the dry sample (g/100g).

### 2.4. Phenolic Extraction

The phenolic extraction was carried out as previously described by Jara-Palacios et al. [27], with some modifications. Briefly, 1 g of each dry sample was homogenized in 15 mL of 75% (*v*/*v*) methanol by agitation for 24 h in an incubated mini shaker (VWR International, Barcelona, Spain) and then centrifuged (3220× *g* for 10 min). The supernatant was collected, and the residue was submitted to the same procedure twice, although it remained in agitation for only 1 h. The supernatants were combined (phenolic extract). The extractions were carried out in triplicate.

### 2.5. Total Phenolic Content (TPC)

The total phenolic concentration (mg/g of dry sample) was analyzed by the Folin–Ciocalteau method detailed by Singleton & Rossi [40]. Briefly, 500 μL of Folin–Ciocalteu reagent, 100 μL of each phenolic extract (1, 1/2 and 1/10 dilutions), and 1.5 mL of 20% (*w*/*v*) Na_2_CO_3_ were mixed in a 10 mL volumetric flask and finally made up to the mark with distilled water. It was incubated in the dark for 2 h at room temperature for the reaction to take place. Then, the absorbance was measured at 765 nm with an Agilent UV-vis HP8453 spectrophotometer (Palo Alto, CA, USA). Gallic acid was used as a calibration standard, and the results were expressed as gallic acid equivalents (mg GAE/g of dry sample).

### 2.6. HPLC-DAD Analysis of Polyphenolic Compounds

HPLC separation, identification, and quantification of the different phenolic compounds of the by-products were obtained. Two mL of each phenolic extract was concentrated to dryness and dissolved in 1 mL of 0.01% (*v*/*v*) formic acid. Samples were prefiltered through a 0.45 μm nylon filter and analyzed in triplicate.

The monomeric anthocyanins, flavonols, and hydroxycinnamic acid derivatives were analyzed using an Agilent 1200 chromatographic system equipped with a quaternary pump and a UV–vis diode array detector, an automatic injector, and the ChemStation B.04.03 version software (Palo Alto, CA, USA). A reversed-phase column Zorbax C18 (250 × 4.6 mm, 5 μm particle size) was used, and the solvents used were as follows: A, acetonitrile–formic acid-water (3:10:87) and B, acetonitrile–formic acid–water (50:10:40). The UV–vis spectra were recorded from 200 to 800 nm with 2.0 nm bandwidth. Individual monomeric anthocyanins were determined according to the method described by Heredia et al. [41]. The flow rate was 0.8 mL/min. The injection volume was 50 μL, and the column was thermostatted at 38 °C. The elution profile was as follows: 0–10 min, 94% A-6% B; 10–15 min, 70% A-30% B; 15–25 min, 60% A-40% B; 25–35 min, 55% A-45% B; 35–40 min, 50% A-50% B; 40–42 min, 40% A-60% B; and 42–43 min, 94% A-6% B. Malvidin-3-glucoside was used as standard for the quantification of the different individual anthocyanins were quantified at 525 nm. For the identification and quantification of individual flavonols and hydroxycinnamic acid derivatives (HCAD), the method of Gordillo, et al. [42] was followed. Fifty μL of sample were injected, and the column was conditioned at 40 °C and with a flow rate of 0.63 mL/min. The elution profile was as follows: 0–5 min, 94% A-6% B; 5–15 min, 89% A-11% B; 15–20 min, 80% A-20%B; 20–25 min, 77% A-23% B; 25–30 min, 74% A-26% B; 30–35 min, 60% A-40% B; 35–38 min, 50% A-50% B; 38–46 min, 40% A-60% B; and 46 min, 94% A-6% B. Quantification of the individual phenolic compounds was performed at 320 nm (HCAD) and 360 nm (flavonols) by comparing the areas and the retention times with the standards of caffeic acid and quercetin, respectively.

Hydroxybenzoic acids, flavan-3-ols, and procyanidins were analyzed following the previously published analysis conditions [27] and using an Agilent 1290 chromatographic system equipped with a quaternary pump, a UV–vis diode array detector, an automatic injector, and ChemStation software (Palo Alto, CA, USA). The UV–vis spectra were recorded from 200 to 770 nm with a 2.0 nm bandwidth. A C18 Eclipse Plus 120 (1.8 μm, 50 × 2.1 mm) column at 25 °C was used at 0.8 mL/min of the solvents (0.01% formic acid as solvent A and acetonitrile as solvent B) with the following gradient: 0–5 min, 5% B; 5–20 min, 50% B; and 20–25 min, 100% A. Injected was 0.5 μL of the sample. Quantification of the individual phenolic compounds was performed at 280 nm by comparing the areas and the retention times with the standards of gallic acid and protocatechuic acid (hydroxybenzoic acids) and (+)-catechin (flavan-3-ols and procyanidins). The results of all phenolic compounds were expressed as µg/g of dry sample.

The total content of each family was calculated as the sum of individual polyphenolic compounds identified by HPLC.

### 2.7. Antioxidant Capacity

#### 2.7.1. DPPH Assay

The ability of the samples to scavenge DPPH• radicals was evaluated according to the method proposed by Soler-Rivas et al. [43]. First, 300 μL of 108 μM DPPH• methanolic solution were added to 30 μL of each phenolic extract (1/10, 1/25, 1/50, and 1/100 dilutions), standard (Trolox), or 80% (*v*/*v*) methanol (blank), and the mixture was diluted with 570 μL of 80% (*v*/*v*) methanol. After 30 min (room temperature in dark conditions), the absorbance was measured on an Agilent UV-vis HP8453 spectrophotometer (Agilent Technologies, Palo Alto, CA, USA) at 515 nm. The antioxidant activity was expressed as μmol TE (Trolox equivalents)/g of dry sample. Measurements were made in triplicate.

#### 2.7.2. ABTS Assay

The ABTS free-radical-scavenging activity of each phenolic extract was determined [44]. A 7 mM ABTS•+ stock solution was prepared using potassium persulfate, 2.45 mM, as the oxidizing agent. After 12–16 h stored in dark conditions and at room temperature, this stock solution was diluted with phosphate buffer to form the test reagent, with an absorbance of 0.700 ± 0.02 at 734 nm. Two mL of this reagent were mixed with 50 μL of each phenolic extract or different concentrations of Trolox standard solution (0.03–1.00 mmol/L). An Agilent UV–vis HP8453 spectrophotometer was used for determining the absorbance at 734 nm after 4 min of reaction. Each phenolic extract achieving 20–80% inhibition of the blank absorbance was selected for the calculations. For this purpose, different dilutions of each phenolic extract were necessary (1/12.5, 1/25, 1/50, 1/100, 1/200, and 1/400). The results were expressed as Trolox equivalents (TE)/g of dry sample. The analyses were carried out in triplicate.

### 2.8. Cyclic Voltammetry

The electrochemical behavior of the samples was evaluated by cyclic voltammetry using a potentiostat/galvanostat (AUTOLAB model PGSTAT 302 N, Metrohm Autolab B.V., Utrecht, The Netherlands) controlled by General Purpose Electrochemical System (GPES) Nova 1.11 software (Metrohm Autolab B.V., Utrecht, The Netherlands) and equipped with a conventional three-electrode system consisting of a glassy carbon working electrode, a platinum auxiliary electrode, and an Ag/AgCl reference electrode. The cyclic voltammogram scans were made from 0.0 to 1.0 V at a scan rate of 100 mV/s [27].

The phenolic extracts were diluted in a 0.1 M sodium acetate–acetic acid buffer at pH 3.6 and transferred into a glass electrochemical cell (EG&G, Princeton, NJ) for the measurements. For each sample, three phenolic extracts were used, and therefore, each sample is associated with three voltammograms.

The electrochemical parameters were obtained from the voltammogram data: the anodic area (Q_anodic_) that represents the integrated area of the cyclic voltammogram for anodic scans taken from 0 to 1 V, the anodic potential and anodic current for two peaks (E_pa_ and I_pa_, respectively), and the cathodic potential and cathodic current for one peak (E_pc_ and I_pc_, respectively).

### 2.9. Statistical Analysis

Statistica v.8.0 software (StatSoft Inc., Tulsa, OK, USA, 2007) was used to perform all statistical analyses. A univariate analysis of variance (ANOVA) was applied to establish the statistical differences between the oenological by-products studied. Additionally, simple and multiple correlations between the contents of phenolic compounds and the antioxidant activity were studied. In all cases, a statistically significant level was considered at *p* < 0.05.

Pattern recognition techniques (PR), like stepwise linear discriminant analysis (SLDA), were applied to the experimental standardized data in order to classify different oenological by-products.

## 3. Results and Discussion

Table 2 and Table 3 summarize the values of % protein, phenolic profile and content, and antioxidant activity using the DPPH and ABTS of all the studied samples. Table 4 shows the ANOVA test according to the studied factors: by-product, type of vinification, variety, and season.

### 3.1. Climatic Conditions

According to the Köppen climatic classification [45], the center and southwest of Spain have a typically warm climate with a “Csa” assignation, according to the classification of the main climates and precipitation and temperature conditions. Thus, “C” refers to “warm temperature,” “s” to “summer dry”, and “a” to “hot summer,” respectively.

In the 2022 and 2023 vintages, respectively, the average temperature was 18.9 °C (range 25.2 to 13.7 °C) and 19.3 °C (range 26.0 °C and 13.8 °C), the humidity was 65.1% (range 87.0 to 40.0%) and 57.9% (range 79.3 to 35.3%), and the rainfall 1.28 and 1.00 mm/day (data provided by Instituto de Investigación y Formación Agraria y Pesquera (IFAPA), Junta de Andalucía, Spain).

### 3.2. Protein Content

The protein content of all samples ranged from 3.96% to 32.24%, which was in concordance with other studies [46,47]. Considering the effect on protein content of the studied factors, lees displayed the significantly (*p* < 0.05) highest content among the types of by-products (Table 2 and Table 4). The autolysis of fermentation yeasts, after cell death, leads to the release of cellular proteins, among other compounds, thus remaining in the lees [48]. Moreover, Pérez-Bibbins et al. [9] affirmed that the solid fraction of lees is a combination of yeasts, organic acids (mainly tartaric acid), insoluble carbohydrates (such as cellulosic or hemicellulosic materials), inorganic salts, lignin, proteins, phenolic compounds, pulp, and other parts of the grape. These results propose the lees as an alternative protein source from a winemaking residue to direct application in other industries, such as cosmetics, biomass, or animal feed.

Regarding the variety factor, it exerted a significant (*p* < 0.05) influence on the percentage of proteins (Table 4), obtaining the by-products coming from Syrah–Tempranillo grape varieties with a significantly (*p* < 0.05) higher content compared to the rest of the varieties. Furthermore, it is remarkable that the percentage of protein was independent of the vinification typology (red, rosè, or white) (Table 4). This fact may be due to the proteins, mainly present in the grape skins and seeds [49], that have low extractability under winemaking conditions and are left in those wine by-products, suggesting that the maceration time of the skins (in red or rosé vinifications) is irrelevant in terms of protein content. Finally, it is noteworthy that the percentage of protein remained constant through the seasons, highlighting the invariable character of this parameter to the climatic conditions.

### 3.3. Phenolic Composition

An extensive characterization of the phenolic profile of the different by-products was carried out (Table 2 and Table 3), including monomeric anthocyanins (non-acylated, acetylated, and *p*-coumaroylated derivatives of delphinidin, cyanidin, petunidin, peonidin, and malvidin), flavonols (3-glucoside and 3-glucuronide derivatives of myricetin, quercetin, laricitrin, kaempherol, isorhamnetin, and syringetin), hydroxycinnamic acid derivatives (trans-caftaric and coutaric acids), hydroxybenzoic acids (gallic and protocatechuic acids), monomeric flavan-3-ols ((+)-catechin, (‒)-epicatechin, epigallocatechin gallate and epicatechin gallate), and procyanidins (B1, B2, B4, and C1, trimer 1 and 2, tetramer 1 and 2, and galloyled procyanidin 1 and 2). All of them have been previously identified in grape pomace [26,50], but this is the first attempt to extensively identify and quantify them in lees and grape seed meal by-products.

Regarding the type of by-product factor (pomace, lees, or seed meal), significant (*p* < 0.05) differences were observed in the content of HACD, hydroxybenzoic acids, flavan-3-ols, and procyanidins (Table 4). HACD displayed a significantly (*p* < 0.05) higher content in lees, followed by grape pomaces, with the seed meal having the least amount (Table 2 and Table 3). This behavior is consistent with the reports of Teixeira-Barcia et al. [51] when grape and winemaking by-products of Brazilian hybrid cultivars have been characterized. Contrarily, lees emerged as the poorest of the hydroxybenzoic acids, flavan-3-ols, and procyanidins, especially galloylated, B1, B2, and C1 procyanidins. These outcomes suggest that yeast cell walls adsorb phenolic compounds, establishing molecular interactions between them, especially hydroxycinnamic acid derivatives, which implies that wine lees could be considered as an important phenolic-rich raw material [52]. No remarkable differences in anthocyanins and flavonols, according to the type of by-product, have been observed.

Concerning the vinification typology factor (red, rosè, or white), as expected, the content of all monomeric anthocyanins was significantly (*p* < 0.05) higher in by-products coming from rosè vinification (Table 2, Table 3 and Table 4). This behavior could be due to the fact that the maceration time is lower in rosè vinifications compared to the red vinification, resulting in the level of most of the anthocyanins being augmented as they remained in the skins [53]. A similar pattern was observed for some flavonols, such as myricetin, laricitrin, and isorhamnetin derivatives. These results differed from those obtained regarding monomeric flavan-3-ols and procyanidins, whose content was significantly (*p* < 0.05) higher in the by-products coming from red vinification type (i.e., more maceration time) (Table 2 and Table 3). This fact could be pertaining to the low extractability of flavan-3-ols and procyanidins [54], compared to other phenolic compounds such as anthocyanins, increasing their content more in by-products from red vinifications as a result of other phenolic compounds being extractable more easily. For all phenolic compounds, by-products from the white vinification typology depicted the lowest level.

The variety factor significantly (*p* < 0.05) influenced the content of phenolic compounds (Table 4). As expected, anthocyanins were only identified in red grape varieties and, among them, the by-products of Tempranillo displayed the highest amount of anthocyanins and procyanidins, as well as some flavonols (myricetin derivatives) and hydroxycinnamic acid derivatives. On the contrary, the by-products derived from white varieties (Airén and Zalema) emerged as the samples with the lowest values of flavonols, hydroxycinnamic acid derivatives, and hydroxybenzoic acids (gallic acid). However, the behavior of each procyanidin considerably differed among them according to the variety factor. Pérez-Bibbins et al. [9] and Rankine et al. [55] affirmed that the composition of lees greatly depends on the grape variety.

Scarce differences in phenolic composition have been observed among the grapevine growing seasons (Table 4), in concordance with the similar climatic conditions previously described. Only the amounts of anthocyanins, some flavonols, the procyanidins B2 and C1, and the galloylated procyanidin1 displayed significantly (*p* < 0.05) higher content in 2022, contrary to the behavior of the procyanidins B4, trimer 1, and tetramers 1 and 2 (Table 3).

### 3.4. Antioxidant Activity

#### 3.4.1. DPPH and ABTS

Table 2 exhibits the values of antioxidant activity of the different by-products. Considering the data of both DPPH and ABTS, it seems that both methodologies followed the same patterns. According to Table 4, the type of by-product significantly (*p* < 0.05) influenced the antioxidant activity of DPPH, with higher values in the grape pomace, followed by the lees and grape seed meal. Concretely, the Tempranillo red and rosè vinifications seemed to be the most antioxidant by-products, whereas Airén white vinification showed the lowest values of antioxidant capacity, both by DPPH and ABTS. In agreement with previous surveys dealing with the direct relation among the antioxidant activity from winemaking by-products and the total content of phenolic compounds [5], the higher antioxidant activity could be supported by the behavior of all phenolic compounds in the Tempranillo variety, whose content was significantly (*p* < 0.05) higher, regardless of the vinification typology and type of by-product. Differences among seasons were only detected when the DPPH assay was used to evaluate the antioxidant capacity, with the highest values in 2022 being possibly related to the high levels of procyanidins, anthocyanins, and some flavonols, which is in agreement with the findings of other authors [56].

In light of the reported relationship between phenols from enological by-products and antioxidant activity [57,58], regression analyses were carried out for both antioxidant capacity methodologies used. In the case of DPPH and ABTS analyses, the correlation coefficients (0.926 and 0.885, respectively) indicated a direct correlation between the total phenolic content (TPC) and the antioxidant activity.

Also, multiple regression analyses were performed to check the more influential non-colored phenolic groups (independent variables: flavonols, hydroxycinnamic acid derivatives, hydroxybenzoic acids, monomeric flavan-3-ols, and procyanidins) on the antioxidant activity (dependent variable: DPPH or ABTS). For the dependent variable DPPH, a high multiple correlation coefficient (R^2^ = 0.85) and a significant correlation (*p* < 0.05) were obtained for the flavonols (β = 0.58), followed by the procyanidins and monomeric flavan-3-ols (β = 0.35 and β = 0.26). When the antioxidant activity measured by ABTS was taken as the dependent variable, a high and significant (*p* < 0.05) multiple correlation coefficient (R^2^ = 0.83) was also obtained, with the independent variables hydroxycinnamic acid derivatives, monomeric flavan-3-ols, and procyanidins having the most weight in the prediction equation (β = 0.56, β = 0.45 and β = 0.37, respectively).

In addition, with the data from the group of samples containing colored phenols (anthocyanins), multiple regression analyses were performed to check which phenolic groups (independent variables: monomeric anthocyanins, flavonols, hydroxycinnamic acid derivatives, hydroxybenzoic acids, monomeric flavan-3-ols and procyanidins) have the greatest influence on the antioxidant activity (dependent variable: DPPH or ABTS). Thus, for the dependent variable DPPH, also, a high multiple correlation coefficient (R^2^ = 0.86) and a significant correlation (*p* < 0.05) were obtained for the monomeric anthocyanins (β = 0.55), followed by the hydroxybenzoic acids, procyanidins, and monomeric flavan-3-ols (β = 0.28, β = 0.24 and β = 0.21, respectively). Finally, when ABTS was taken as a dependent variable, a higher and significant (*p* < 0.05) multiple correlation coefficient (R^2^ = 0.90) was obtained, and a significant correlation (*p* < 0.05) were obtained for the hydroxycinnamic acid derivatives (β = 0.60) followed by monomeric flavan-3-ols, procyanidins, and monomeric anthocyanins (β = 0.45, β = 0.40 and β = 0.32 respectively).

#### 3.4.2. Cyclic Voltammetry

**Figure 1:**
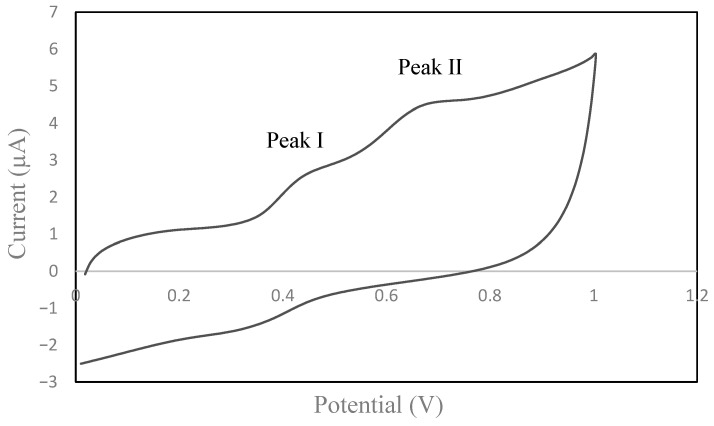
Representative cyclic voltammogram of grape pomace.

Cyclic voltammetry was used to evaluate the electrochemical behavior of the different by-products. The cyclic voltammograms of the samples gave a set of anodic (positive) and cathodic (negative current) peaks. In Figure 1, a typical voltammogram corresponding to a grape pomace can be observed.

The electrochemical parameters, that describe the redox process, were extracted from the voltammograms of the by-product extracts, and the values are shown in Table 5. The parameters E_pa_ and E_pc_ are related to the nature of the antioxidant compounds (qualitative analysis), and I_pa_, I_pc_, and Q are related to the concentration of these compounds (quantitative analysis).

For all the by-products, two anodic peaks were considered. No peak at about 0.2 V has been considered because the initial current increase might not be related to the phenolic compounds. In previous studies, the peak at 0.2 V was attributed to phenolic compounds containing a flavonoid structure with a catechol or a galloyl group (i.e., ortho-diphenol and triphenol groups) at the B-ring [18,25,59]. In this study, the two anodic peaks, namely peak I and peak II, ranged between 0.406 and 0.471 V and between 0.641 and 0.697 V, respectively. Peak I could correspond to the irreversible oxidation of the -OH group at position 3 on the C-ring [60] being flavanols such as catechin, which are able to produce this peak [25], and flavonols, which are derivatives of quercetin [59]. Peak II could be due to phenolic acids, flavanols, and flavonols [25,61]. On the reverse scan, as expected, only one peak is depicted, between 0.317 and 0.373 V. As previously reported, this peak could be related to a reversible electrode reaction, which is typical of ortho-diphenol compounds [18]. The Q_anodic_ can be used as a measure of the total antioxidant potential related to the concentration of total phenolic compounds. A major difference between the types of by-products was found for the Q_anodic_ values (between 1.40–2.65 units).

In general, different electrochemical behavior indicates differences in antioxidant activity and, therefore, in the type and quantity of the phenolic compounds. For this purpose, an ANOVA test was made to establish significant differences between the samples in relation to the electrochemical parameters.

**Figure 2:**
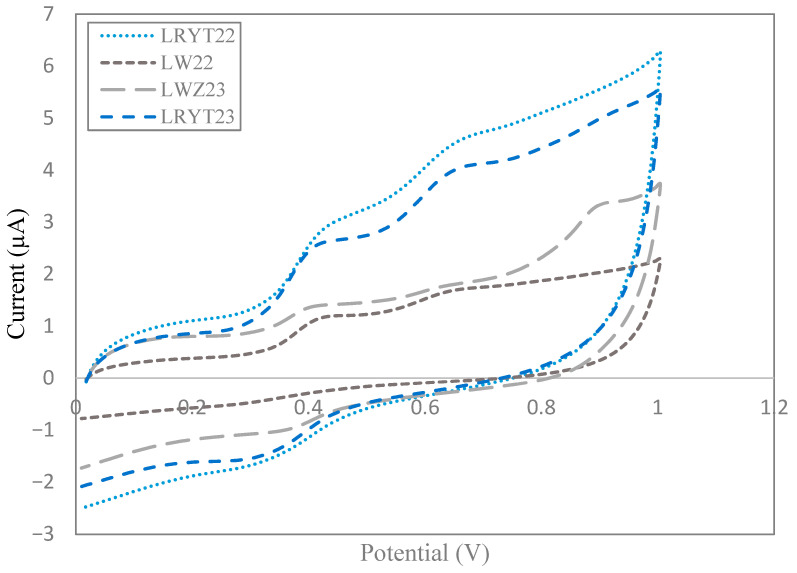
Cyclic voltammogram of white and red lees.

Regarding the type of by-product, on the one hand, significant differences (*p* < 0.05) were found for E_pa1_ and for E_pa2_ between the lees and grape pomaces, which is indicative that the qualitative phenolic profile of these types of by-products is different. On the other hand, some significant differences (*p* < 0.05) were found in quantitative terms: White lees showed lower values (*p* < 0.05) for parameter I_pa1_ than red lees, seed meal, and almost all samples of pomace (Table 5). For I_pa2_, white lees also had lower values (*p* < 0.05) than red lees and grape pomaces, and the seed meal differed significantly with almost all the pomace samples. These differences in the electrochemical parameters between the samples are due to the different amounts of the different types of identified phenolic compounds. The parameter Q_anodic_, related to the total phenol content, indicates significant differences (*p* < 0.05) between white lees and red lees (Figure 2), as well as with most of the grape pomaces.

**Figure 3:**
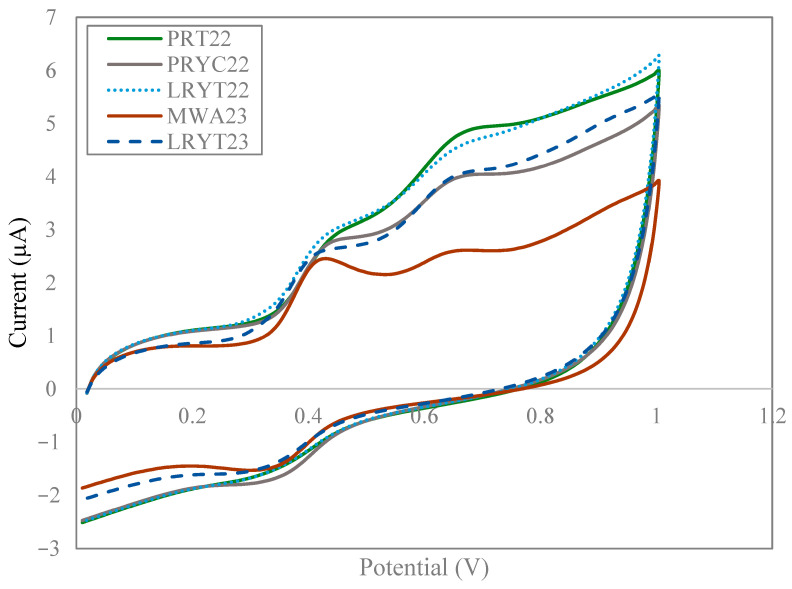
Cyclic voltammogram of seed meal compared to red lees and two grape pomaces.

There are also significant differences (*p* < 0.05) between seed meal with red lees and some grape pomaces (Figure 3). In general, the lowest Q_anodic_ values are for white lees (between 1.40 and 1.73 units) and the highest for red lees and Tempranillo grape pomaces (between 2.29 and 2.65 units), with these differences being significant (*p* < 0.05).

Considering the vinification typology factor, the electrochemical parameter I_pa2_ showed significant differences (*p* < 0.05) between the by-products coming from white and rosè vinifications and I_pa1_, I_pa2_, and Q_anodic_ between the by-products coming from white and red vinifications.

Taking into account the variety factor, significant differences (*p* < 0.05) were found between the white and red varieties for two quantitative electrochemical parameters, namely I_pa2_ and Q_anodic_, which differed between the white varieties (Zalema and Airén) and Tempranillo. This is due to the different compositions between white and red varieties, mainly due to the anthocyanin content.

Finally, the grapevine growing seasons influenced the values of all the electrochemical parameters, and significant differences were found for all of them, except for E_pc_.

### 3.5. Linear Discriminant Analysis

To ascertain whether it was possible to discriminate between different types of by-products, different vinification typologies, and different varieties, SLDA analyses were performed based on the protein content, total phenolic content (TPC), phenolic composition (total monomeric anthocyanins, total flavonols, total HACD, total hydroxybenzoic acids, total monomeric flavan-3-ols, and total procyanidins), DPPH, ABTS, and electrochemical parameters by cyclic voltammetry (I_pa1_ and I_pa2_) (independent variables).

**Figure 4:**
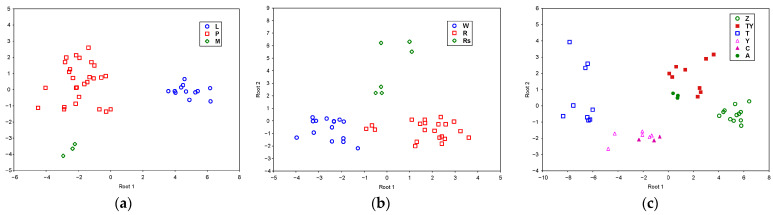
Scatterplot of the by-products samples in the plane defined by the canonical function when protein content, phenolic composition, and antioxidant activity are considered for discrimination. (**a**) Type of by-products (L: lees, P: pomace, M: seed meal), (**b**) vinification typology (W: white, R: red, Rs: rosè), (**c**) variety (Z: Zalema, TY: Syrah–Tempranillo 50:50, T: Tempranillo, Y: Syrah, C: Syrah chips, A: Airén).

The mathematical model obtained for the different by-products (lees, pomace, and seed meal) included 4 of the 12 variables: protein content, total hydroxybenzoic acids, TPC, and total HACD. Two classification functions were obtained, with a 100% correct classification of the samples on their own. Discriminant function 1 is mainly related to protein content (with a positive sign) and total hydroxybenzoic acids (with a negative sign), whereas discriminant function 2 is mainly linked to TPC (with a positive sign) and total hydroxybenzoic acids (with a negative sign). As can be seen in Figure 4a, discriminant function 1 discriminates mainly between lees and the other by-products, and discriminant function 2 between grape seed meal and the other by-products.

In the SLDA for different vinifications (white, red, and rosè), the variables included were six: total monomeric anthocyanins, protein content, total hydroxybenzoic acids, total flavonols, TPC, and DPPH. Two classification functions were obtained, with a 100% correct classification of the samples. Discriminant function 1 is related to TFC (with a positive sign) and total flavonols and DPPH (with a negative sign), and discriminant function 2 is mainly linked to total monomeric anthocyanins (with a positive sign), protein content, and DPPH (with a negative sign). Discriminant function 1 discriminates mainly between white vinification and the other typologies and discriminant function 2 between rosè and the other vinification typologies (Figure 4b).

To discriminate between different varieties, the mathematical model obtained included 6 of the 12 independent variables: total monomeric anthocyanins, protein content, total monomeric flavan-3-ols, total flavonols, total procyanidins, and TPC. The location of the samples within the plane defined by the two canonical functions showed a good separation (98% correct classification) as a function of the variety (Figure 4c). Function 1 allowed the samples to be separated into two categories, namely T, Y and C, clearly located on the left area of the graphic, with Z, A, and TY on the right. This differentiation was related to the total flavonols (taking a positive sign) and total monomeric anthocyanins (taking a negative sign). Discriminant function 2 was linked to the total procyanidins and protein content (with a positive sign) and TPC (with a negative sign).

Although all winemaking by-products are susceptible to the extraction of compounds of interest and should be considered for revalorization, some are better sources than others. Thus, if the goal is to extract proteins and hydroxycinnamic acids from winemaking by-products, the optimal product to use would be red wine lees [51,52]. However, to obtain phenolic compounds (especially anthocyanins, flavan-3-ols, and procyanidins), the best by-products to consider are those derived from rosé winemaking (grape pomace), particularly from the Tempranillo variety [53], which is associated with a high contribution to antioxidant capacity [57,58].

This study represents the first comprehensive approach to addressing multiple factors involved in the revalorization of winemaking by-products, offering valuable insights for the wine and grape pomace industries, whose extracted compounds hold potential for application in cosmetics, pharmaceuticals, and other high-value sectors.

## 4. Conclusions

The different factors addressed, i.e., type of by-product, type of winemaking, variety, and season of the year, which influence the chemical composition and consequently the antioxidant activity of the by-products, have not been previously analyzed, and even less with an in-depth identification and quantification of phenolic compounds, together with the evaluation of their antioxidant profile by voltammetry. Likewise, although many studies on grape pomace have been published in the bibliography, the potential of lees and grape seed meal is still not very extensive from the point of view of its revaluation. Based on the considerable amount of non-extracted antioxidant phenols and proteins in the by-products and residues studied, it is evident that the wine industry could revalorize them, obtaining an economic benefit by extracting them in its facilities or by selling them to other industries for use in other purposes, such as biostimulants or biofertilizers. Therefore, this work is a step forward for reusing winemaking by-products in industries such as agriculture, cosmetics, pharmaceuticals, or functional foods, among others.

## Figures and Tables

**Table 1:** Nomenclature and description of the analyzed samples.

	Type By-Product	Vinification Typology	Variety	Season
PRsT22	Pomace	Rosè	Tempranillo	2022
PRsY22	Pomace	Rosè	Syrah	2022
PRT22	Pomace	Red	Tempranillo	2022
PRYC22	Pomace	Red	Syrah chips	2022
PRY22	Pomace	Red	Syrah	2022
PWZ22	Pomace	White	Zalema	2022
LRYT22	Lees	Red	Syrah:Tempranillo 50:50	2022
LWZ22	Lees	White	Zalema	2022
PRYT23	Pomace	Red	Syrah:Tempranillo 50:50	2023
PRT23	Pomace	Red	Tempranillo	2023
PWZ23	Pomace	White	Zalema	2023
LRYT23	Lees	Red	Syrah:Tempranillo 50:50	2023
LWZ23	Lees	White	Zalema	2023
MWA23	Seed meal	White	Airén	2023

**Table 2:** Mean values (and standard deviations, in brackets) (*n* = 3) of protein content (%), total monomeric anthocyanins, flavonols, hydroxycinnamic acid derivatives (HACD), hydroxybenzoic acids, monomeric flavan-3-ols, procyanidins (µg/g), total phenolic content (TPC) (mg/g), and antioxidant capacity (DPPH and ABTS) (µmoles TE/g) of the samples.

	PRsT22	PRsY22	PRT22	PRYC22	PRY22	PWZ22	LRYT22	LWZ22	PRYT23	PRT23	PWZ23	LRYT23	LWZ23	MWA23
Protein content	3.96	5.12	12.47	10.49	12.21	9.59	32.24	15.74	12.24	12.57	8.86	20.44	13.92	11.72
	(0.01)	(0.04)	(0.55)	(1.29)	(2.18)	(0.77)	(0.98)	(1.53)	(0.88)	(1.49)	(0.81)	(0.36)	(0.78)	(0.72)
Total monomeric anthocyanins	5090.18	1354.16	3447.12	1080.96	2053.06	nd	3982.77	nd	134.12	1691.74	nd	1976.47	nd	nd
	(682.80)	(78.37)	(367.60)	(71.10)	(93.78)		(212.35)		(4.87)	(292.46)		(93.65)		
Total flavonols	418.90	113.90	297.89	121.93	217.92	726.84	416.08	nd	96.10	148.47	422.12	225.35	nd	24.88
	(58.99)	(3.46)	(44.84)	(20.90)	(25.44)	(36.51)	(7.93)		(7.82)	(15.22)	(33.29)	(33.18)		(1.00)
Total HACD	376.15	99.95	82.08	57.71	66.74	67.53	235.77	90.77	6.21	97.58	17.77	1177.67	0.63	5.88
	(14.34)	(17.09)	(1.59)	(12.39)	(9.95)	(3.87)	(18.55)	(28.76)	(0.99)	(3.88)	(3.32)	(213.51)	(0.07)	(1.17)
Total hydroxybenzoic acids	261.65	159.29	463.63	305.30	320.28	293.14	327.50	nd	233.66	291.38	312.08	260.74	nd	364.95
	(29.73)	(15.92)	(77.87)	(39.98)	(29.52)	(26.88)	(28.37)		(4.07)	(12.82)	(23.16)	(14.10)		(39.49)
Total monomeric flavan-3-ols	554.48	443.71	720.44	732.98	1130.41	860.90	619.25	nd	326.36	1257.91	548.93	415.59	273.47	690.61
	(105.15)	(33.54)	(93.00)	(65.71)	(82.25)	(67.77)	(57.27)		(6.53)	(124.76)	(25.03)	(16.10)	(6.94)	(56.07)
Total procyanidins	1199.22	704.06	2473.68	755.32	877.44	2397.97	585.39	nd	1119.89	1726.34	1223.57	1463.59	118.55	1174.14
	(64.93)	(76.27)	(132.19)	(56.47)	(106.26)	(220.58)	(54.48)		(45.87)	(46.17)	(62.61)	(121.17)	(2.56)	(62.27)
TPC	32.64	16.29	35.71	23.80	29.09	34.66	37.88	7.06	11.56	32.31	16.55	32.73	4.11	4.77
	(1.49)	(0.69)	(2.52)	(1.10)	(1.11)	(3.24)	(3.49)	(0.75)	(0.58)	(1.63)	(0.83)	(0.35)	(0.31)	(0.67)
DPPH	229.53	79.02	330.59	123.84	186.49	332.95	244.53	7.84	34.53	211.37	100.07	200.01	18.38	41.95
	(42.02)	(21.44)	(28.03)	(9.52)	(2.42)	(15.03)	(10.01)	(0.82)	(2.30)	(47.61)	(1.91)	(14.93)	(0.68)	(2.62)
ABTS	404.53	157.22	490.81	215.59	293.76	424.32	362.84	79.77	71.23	529.03	204.93	557.89	89.75	105.23
	(93.30)	(19.09)	(28.62)	(2.91)	(5.88)	(43.02)	(43.60)	(5.59)	(5.49)	(43.09)	(20.36)	(32.74)	(2.03)	(33.53)

HACD, hydroxycinnamic acid derivatives; TPC, total phenolic content.

**Table 3:** Mean values (and standard deviations, in brackets) (*n* = 3) of individual monomeric anthocyanins, flavonols, hydroxycinnamic acid derivatives (HACD), hydroxybenzoic acids, monomeric flavan-3-ols, and procyanidins (µg/g) of the samples.

	PRsT22	PRsY22	PRT22	PRYC22	PRY22	PWZ22	LRYT22	LWZ22	PRYT23	PRT23	PWZ23	LRYT23	LWZ23	MWA23
*Monomeric anthocyanins*														
Delphinidin-3-glucoside	387.74	73.62	146.10	59.87	66.45	nd	138.66	nd	nd	62.03	nd	98.72	nd	nd
	(77.34)	(6.48)	(14.15)	(0.92)	(10.01)		(1.96)			(2.85)		(4.46)		
Cyanidin-3-glucoside	95.32	58.66	61.60	0.00	0.00	nd	59.67	nd	nd	61.04	nd	60.99	nd	nd
	(10.46)	(3.08)	(2.56)	(0.00)	(0.00		(2.66)			(3.06)		(4.09)		
Petunidin-3-glucoside	400.93	86.63	215.61	62.66	85.41	nd	184.24	nd	nd	63.66	nd	131.11	nd	nd
	(70.92)	(10.50)	(25.01)	(1.72)	(20.73)		(5.64)			(8.04)		(6.87)		
Peonidin-3-glucoside	188.82	86.75	102.61	73.23	105.63	nd	114.36	nd	nd	136.13	nd	77.21	nd	nd
	(25.98)	(13.02)	(5.22)	(7.32)	(12.66)		(8.42)			(14.39)		(4.22)		
Malvidin-3-glucoside	1558.75	276.27	1118.87	139.30	437.47	nd	980.28	nd	69.68	482.13	nd	620.57	nd	nd
	(256.23)	(3.50)	(148.25)	(17.53)	(58.42)		(17.76)		(2.78)	(87.01)		(32.06)		
Petunidin-3-acetyl-glucoside	127.81	74.01	96.70	71.79	81.97	nd	89.82	nd	nd	66.07	nd	89.67	nd	nd
	(12.71)	(6.08)	(6.82)	(16.22)	(23.97)		(2.39)			(2.09)		(6.50)		
Peonidin-3-acetyl-glucoside	78.35	63.07	66.91	68.72	157.53	nd	65.39	nd	nd	66.13	nd	70.64	nd	nd
	(5.42)	(1.04)	(4.39)	(4.65)	(19.30)		(6.60)			(5.18)		(1.68)		
Malvidin-3-acetyl-glucoside	312.11	170.48	212.94	119.17	295.00	nd	274.26	nd	nd	102.12	nd	232.39	nd	nd
	(40.48)	(4.35)	(35.87)	(5.22)	(25.85)		(29.02)			(15.18)		(8.13)		
Delphinidin-3-*p*-coumaroyl-glucoside	248.55	69.49	200.03	84.24	109.80	nd	237.59	nd	nd	73.25	nd	88.06	nd	nd
	(34.26)	(7.65)	(48.89)	(12.55)	(28.39)		(3.13)			(8.85)		(3.60)		
Petunidin-3-*p*-coumaroyl-glucoside	148.37	75.89	120.49	82.46	103.23	nd	110.64	nd	nd	74.13	nd	74.62	nd	nd
	(19.06)	(12.13)	(20.07)	(9.31)	(11.61)		(9.85)			(7.54)		(3.09)		
Peonidin-3-*p*-coumaroyl-glucoside	125.91	75.10	100.85	70.59	97.59	nd	183.05	nd	nd	109.42	nd	72.89	nd	nd
	(8.36)	(11.84)	(15.13)	(0.47)	(3.74)		(8.31)			(24.41)		(2.35)		
Malvidin-3-*p*-coumaroyl-glucoside	1192.73	176.48	828.75	163.80	439.72	nd	1308.01	nd	64.44	320.13	nd	266.43	nd	nd
	(119.56)	(12.89)	(35.55)	(33.11)	(39.24)		(111.47)		(2.22)	(119.36)		(22.87)		
*Flavonols*														
Myricetin-3-glucuronide	56.52	6.62	28.04	2.27	2.24	nd	39.82	nd	5.18	nd	9.23	38.86	nd	nd
	(9.22)	(0.36)	(4.58)	(0.02)	(0.08)		(1.08)		(1.04)		(1.19)	(5.14)		
Myricetin-3-glucoside	115.52	16.26	71.32	10.30	15.26	nd	124.33	nd	8.26	nd	nd	37.57	nd	nd
	(14.59)	(0.55)	(8.68)	(1.85)	(4.05)		(2.55)		(0.62)			(3.84)		
Quercetin-3-glucuronide	48.23	12.32	29.52	43.98	51.50	347.23	34.75	nd	21.89	90.04	226.02	114.02	nd	7.34
	(12.53)	(0.91)	(2.93)	(8.07)	(6.83)	(13.01)	(0.98)		(3.70)	(10.93)	(15.59)	(19.05)		(1.14)
Quercetin-3-glucoside	81.31	29.31	59.59	36.23	47.52	301.35	96.11	nd	22.75	15.67	122.48	nd	nd	6.86
	(11.42)	(1.49)	(11.65)	(7.27)	(9.09)	(19.04)	(2.87)		(1.66)	(0.73)	(10.76)			(0.39)
Laricitrin-3-glucoside	62.49	27.95	59.48	2.27	20.16	nd	71.79	nd	3.60	6.45	13.46	nd	nd	0.00
	(7.33)	(1.31)	(9.03)	(0.02)	(0.41)		(1.28)		(0.18)	(0.74)	(1.16)			0.00
Kaempherol-3-glucoside	28.70	14.69	25.71	17.23	6.26	nd	36.16	nd	9.40	4.08	39.89	6.88	nd	0.00
	(3.71)	(3.34)	(3.60)	(3.07)	(1.07)		(0.60)		(0.92)	(1.00)	(4.51)	(2.57)		0.00
Isorhamnetin-3-glucoside	7.74	4.61	7.87	4.90	34.92	nd	10.91	nd	9.27	8.54	3.04	nd	nd	0.00
	(0.58)	(0.14)	(1.68)	(0.86)	(6.51)		(0.14)		(1.15)	(0.83)	(0.14)			0.00
Syringetin-3-glucoside	18.40	2.15	16.35	4.76	40.06	78.26	2.21	nd	15.76	23.70	8.01	28.02	nd	10.67
	(1.91)	(0.08)	(2.71)	(0.02)	(4.05)	(4.72)	(0.10)		(1.12)	(3.07)	(0.50)	(4.01)		(1.09)
*HACD*														
*trans*-Caftaric acid	216.40	76.97	64.97	44.23	55.73	52.02	150.28	85.66	nd	66.67	nd	901.50	nd	nd
	(13.04)	(13.54)	(0.00)	(7.94)	(8.11)	(4.23)	(13.25)	(25.48)		(2.95)		(230.21)		
*trans*-Coutaric acid	159.75	22.99	17.12	13.48	11.00	15.52	85.49	5.11	6.21	30.91	17.77	276.17	0.63	5.88
	(23.84)	(3.58)	(1.59)	(4.64)	(2.15)	(1.72)	(5.29)	(5.43)	(0.99)	(0.93)	(3.32)	(31.99)	(0.07)	(1.17)
*Hydroxybenzoic acids*														
Gallic acid	204.89	159.29	400.34	242.95	258.67	223.38	268.73	nd	176.96	237.89	240.91	260.74	nd	290.03
	(28.86)	(15.92)	(76.11)	(37.88)	(26.03)	(25.79)	(27.96)		(3.76)	(13.58)	(17.67)	(14.10)		(36.18)
Protocatechuic acid	56.76	nd	63.30	62.35	61.61	69.75	58.77	nd	56.69	53.49	71.17	nd	nd	74.92
	(0.90)		(1.76)	(4.51)	(3.49)	(1.09)	(2.31)		(0.94)	(0.95)	(5.54)			(3.33)
*Monomeric flavan-3-ols*														
(+)-catechin	160.38	102.62	161.58	189.34	260.68	272.66	150.41	nd	111.69	445.71	159.18	118.28	128.76	244.55
	(67.90)	(14.49)	(45.25)	(15.43)	(18.30)	(26.03)	(11.96)		(3.54)	(46.69)	(12.14)	(11.59)	(1.69)	(25.13)
(-)-epicatechin	142.33	135.34	120.65	221.24	349.72	187.69	112.19	nd	106.48	476.02	134.74	154.58	144.71	182.51
	(8.11)	(17.39)	(19.80)	(25.75)	(39.67)	(12.89)	(4.69)		(3.31)	(50.20)	(6.15)	(2.89)	(5.38)	(13.12)
Epigallocatechin gallate	130.91	104.80	243.87	322.41	520.01	254.02	356.65	nd	108.20	188.18	135.37	142.73	nd	129.55
	(12.47)	(4.25)	(22.45)	(40.57)	(44.45)	(28.27)	(41.85)		(0.21)	(24.57)	(6.10)	(7.39)		(6.77)
Epicatechin gallate	120.87	100.95	194.34	nd	nd	146.52	nd	nd	nd	147.99	119.64	nd	nd	134.00
	(29.86)	(6.55)	(7.50)			(18.46)				(5.56)	(10.64)			(12.30)
*Procyanidins*														
Procyanidin B1	236.06	146.18	346.11	223.18	240.65	528.74	175.14	nd	115.42	234.33	240.76	148.13	118.55	156.08
	(33.40)	(40.46)	(67.89)	(24.10)	(23.24)	(71.51)	(16.44)		(2.23)	(10.39)	(13.13)	(29.57)	(2.56)	(12.87)
Trimer1	nd	nd	342.93	nd	nd	233.90	nd	nd	215.35	171.44	165.82	254.96	nd	151.87
			(24.91)			(18.33)			(10.22)	(10.07)	(8.76)	(40.36)		(8.55)
Tetramer1	nd	nd	111.11	nd	nd	123.46	nd	nd	99.32	133.57	104.24	206.13	nd	157.65
			(6.77)			(3.01)			(3.20)	(5.66)	(4.44)	(11.95)		(9.17)
Procyanidin B4	nd	nd	nd	nd	nd	223.58	nd	nd	146.45	177.21	113.83	169.45	nd	nd
						(20.22)			(3.20)	(5.30)	(4.17)	(8.12)		
Trimer2	nd	nd	114.44	nd	nd	141.68	107.99	nd	97.42	105.24	115.55	104.90	nd	101.21
			(5.30)			(8.51)	(5.81)		(2.62)	(3.91)	(4.28)	(1.21)		(4.00)
Procyanidin B2	174.48	136.68	218.80	167.99	241.56	253.11	134.72	nd	110.74	226.82	129.92	102.79	nd	119.00
	(8.22)	(10.37)	(10.99)	(20.37)	(17.72)	(21.95)	(18.34)		(0.56)	(16.15)	(7.41)	(2.40)		(9.82)
Galloyled procyanidin1	122.15	nd	107.04	127.87	159.38	132.82	167.53	nd	32.59	128.56	nd	nd	nd	nd
	(7.17)		(3.29)	(2.76)	(34.15)	(6.66)	(16.21)		(56.45)	(6.36)				
Galloyled procyanidin2	161.96	115.31	115.69	nd	nd	147.92	nd	nd	95.55	115.96	101.97	110.93	nd	112.17
	(19.65)	(14.00)	(5.71)			(12.48)			(4.06)	(1.36)	(3.17)	(5.27)		(5.85)
Procyanidin C1	330.58	190.23	407.91	236.28	235.85	342.55	nd	nd	105.59	250.62	123.64	172.31	nd	144.10
	(32.55)	(26.32)	(19.29)	(21.59)	(35.81)	(41.17)			(3.18)	(12.44)	(7.71)	(16.44)		(9.79)
Tetramer2	174.00	115.65	709.63	nd	nd	270.21	nd	nd	101.46	182.59	127.83	194.00	nd	98.06
	(12.47)	(6.48)	(27.61)			(36.38)			(8.92)	(5.06)	(31.18)	(20.57)		(2.41)

**Table 4:** ANOVA analysis applied to all the studied parameters.

	Type of By-Product		Type of Vinification		Variety		Season	
	F	*p*	F	*p*	F	*p*	F	*p*
Protein content	21.4869	0.0000 *	10.2207	0.0003	6.9090	0.0001 *	0.0704	0.7922
Delphinidin-3-glucoside	1.1621	0.3234	21.7220	0.0000 *	7.4659	0.0001 *	7.5627	0.0089 *
Cyanidin-3-glucoside	1.1070	0.3407	24.7429	0.0000 *	15.9574	0.0000 *	1.7532	0.1930
Petunidin-3-glucoside	1.1359	0.3315	19.9067	0.0000 *	8.8995	0.0000 *	8.8933	0.0049 *
Peonidin-3-glucoside	2.8097	0.0725	38.5572	0.0000 *	25.3454	0.0000 *	7.1646	0.0107 *
Malvidin-3-glucoside	1.1562	0.3252	15.9904	0.0000 *	14.7451	0.0000 *	6.5419	0.0144 *
Petunidin-3-acetyl-glucoside	2.3111	0.1126	46.1809	0.0000 *	19.1250	0.0000 *	10.2036	0.0027 *
Peonidin-3-acetyl-glucoside	2.6798	0.0812	24.0100	0.0000 *	19.0462	0.0000 *	9.1005	0.0044 *
Malvidin-3-acetyl-glucoside	1.6964	0.1966	29.6938	0.0000 *	12.0466	0.0000 *	11.9453	0.0013 *
Delphinidin-3-*p*-coumaroyl-glucoside	1.2966	0.2850	17.5439	0.0000 *	8.9484	0.0000 *	14.2096	0.0005 *
Petunidin-3-*p*-coumaroyl-glucoside	2.6015	0.0870	39.1970	0.0000 *	19.9935	0.0000 *	14.5569	0.0005 *
Peonidin-3-*p*-coumaroyl-glucoside	1.6985	0.1962	27.1266	0.0000 *	11.6200	0.0000 *	9.2703	0.0041 *
Malvidin-3-*p*-coumaroyl-glucoside	0.9924	0.3799	11.0346	0.0002 *	7.0980	0.0001 *	10.7318	0.0022 *
*Total monomeric anthocyanins*	1.3740	0.2651	21.0211	0.0000 *	12.0318	0.0000 *	10.3194	0.0026 *
Myricetin-3-glucuronide	1.5271	0.2299	7.7824	0.0014 *	6.2504	0.0003 *	2.1205	0.1531
Myricetin-3-glucoside	1.1881	0.3156	8.3101	0.0010 *	5.2362	0.0010 *	9.2332	0.0042 *
Quercetin-3-glucuronide	2.4956	0.0955	2.6274	0.0850	2.2018	0.0755	0.0338	0.8552
Quercetin-3-glucoside	3.1057	0.0560	1.6033	0.2142	1.4793	0.2207	5.3475	0.0260 *
Laricitrin-3-glucoside	1.0254	0.3681	8.7682	0.0007 *	4.4619	0.0029 *	15.3702	0.0003 *
Kaempherol-3-glucoside	2.5301	0.0927	2.4320	0.1011	1.3702	0.2584	2.2918	0.1379
Isorhamnetin-3-glucoside	3.5373	0.0387 *	6.9775	0.0026 *	6.4709	0.0002 *	4.5496	0.0391 *
Syringetin-3-glucoside	2.7646	0.0754	0.4528	0.6392	0.4303	0.8244	0.8467	0.3630
*Total flavonols*	3.6125	0.0364 *	0.1341	0.8749	1.2906	0.2895	5.1079	0.0293 *
*trans*-Caftaric acid	4.9941	0.0117 *	2.1134	0.1345	2.9531	0.0247 *	0.8802	0.3538
*trans*-Coutaric acid	3.2407	0.0499 *	3.6986	0.0338 *	3.9647	0.0058 *	0.3818	0.5401
*Total HACD*	4.7227	0.0146 *	2.4244	0.1018	3.2903	0.0151 *	0.7664	0.3866
Gallic acid	6.3135	0.0042 *	6.6547	0.0033 *	4.5655	0.0025 *	0.3133	0.5788
Protocatechuic acid	16.8666	0.0000 *	1.4405	0.2491	2.0386	0.0964	0.1733	0.6794
*Total hydroxybenzoic acids*	9.3103	0.0005 *	5.2796	0.0094 *	4.0609	0.0050 *	0.3158	0.5773
(+)-catechin	6.3862	0.0040 *	1.5540	0.2242	2.3240	0.0629	1.4620	0.2337
(-)-epicatechin	4.1560	0.0231 *	3.5513	0.0383 *	2.6991	0.0359 *	1.3736	0.2481
Epigallocatechin gallate	2.5339	0.0923	10.2250	0.0003 *	3.4604	0.0118 *	9.9943	0.0030 *
Epicatechin gallate	12.2647	0.0001 *	2.0550	0.1417	12.2232	0.0000 *	0.0217	0.8836
*Total monomeric flavan-3-ols*	8.8587	0.0007 **	3.8254	0.0304 *	3.3509	0.0138 *	0.2105	0.6489
Procyanidin B1	8.3814	0.0009	0.0640	0.9381	1.1236	0.3655	3.3322	0.0754
Trimer1	1.3179	0.2794	3.6109	0.0364 *	2.8841	0.0273 *	6.1974	0.0171 *
Tetramer1	2.9224	0.0657	3.3437	0.0457 *	4.2311	0.0040 *	23.3446	0.0000 *
Procyanidin B4	1.4147	0.2552	1.8387	0.1725	2.2713	0.0680	**9.5185**	0.0037 *
Trimer2	0.8589	0.4315	5.3686	0.0087 *	5.3208	0.0009 *	6.3720	0.0157 *
Procyanidin B2	22.5022	0.0000 *	4.4378	0.0183 *	3.8705	0.0066 *	4.8810	0.0329 *
Galloyled procyanidin1	4.1695	0.0229 *	6.9101	0.0027 *	3.3756	0.0133 *	16.5583	0.0002 *
Galloyled procyanidin2	7.4004	0.0019 *	4.3391	0.0199 *	3.9339	0.0060 *	1.3416	0.2536
Procyanidin C1	21.2518	0.0000 *	3.3544	0.0453 *	7.3258	0.0001 *	5.0077	0.0309 *
Tetramer2	2.7007	0.0797	0.6470	0.5291	5.0278	0.0014 *	0.5261	0.4725
*Total procyanidins*	7.7590	0.0015 *	1.1378	0.3309	2.6852	0.0367 *	0.0015	0.9691
TPC	5.2927	0.0093 *	10.6994	0.0002	6.3306	0.0003 *	8.4611	0.0059 *
DPPH	3.4099	0.0432 *	3.2896	0.0478	3.4061	0.0127 *	8.3582	0.0062 *
ABTS	2.0060	0.1482	5.7148	0.0067	5.9640	0.0004 *	0.6494	0.4251

Asterisks denote significant differences at *p* < 0.05; HACD, hydroxycinnamic acid derivatives; TPC, total phenolic content.

**Table 5:** Mean values (and standard deviations, in brackets) (*n* = 3) of electrochemical parameters extracted from the cyclic voltammetry curves of the samples.

	E_pa1_	I_pa1_	E_pa2_	I_pa2_	E_pc1_	I_pc1_	Q_anodic_
PRsT22	0.43	2.48	0.67	4.49	0.34	−1.49	2.49
	(0.00)	(0.16)	(0.00)	(0.18)	(0.00)	(0.08)	(0.10)
PRsY22	0.44	2.06	0.67	3.28	0.33	−1.38	1.99
	(0.00)	(0.28)	(0.00)	(0.27)	(0.00)	(0.13)	(0.14)
PRT22	0.42	2.64	0.68	4.89	0.32	−1.62	2.65
	(0.00)	(0.28)	(0.00)	(0.26)	(0.00)	(0.13)	(0.14)
PRYC22	0.43	2.71	0.66	3.98	0.34	−1.69	2.31
	(0.00)	(0.45)	(0.00)	(0.41)	(0.00)	(0.22)	(0.23)
PRY22	0.44	2.68	0.66	4.34	0.34	−1.57	2.44
	(0.00)	(0.32)	(0.00)	(0.26)	(0.00)	(0.16)	(0.12)
PWZ22	0.47	2.58	0.69	4.17	0.33	−1.39	2.31
	(0.00)	(0.20)	(0.00)	(0.26)	(0.00)	(0.07)	(0.11)
LRYT22	0.42	2.83	0.67	4.59	0.34	−1.52	2.64
	(0.00)	(0.31)	(0.00)	(0.28)	(0.00)	0.14	(0.16)
LWZ22	0.42	1.71	0.65	2.15	0.33	−0.42	1.73
	(0.00)	()0.12	(0.00)	(0.22)	(0.00)	(0.08)	(0.14)
PRYT23	0.42	1.66	0.65	2.15	0.33	−1.18	1.49
	(0.00)	(0.02)	(0.00)	(0.31)	(0.00)	(0.03)	(0.11)
PRT23	0.43	2.49	0.67	4.51	0.36	−1.25	2.39
	(0.00)	(0.25)	(0.00)	(0.09)	(0.00)	(0.07)	(0.05)
PWZ23	0.41	2.31	0.64	3.19	0.32	−1.65	2.01
	(0.00)	(0.65)	(0.00)	(0.68)	(0.00)	(0.26)	(0.27)
LRYT23	0.41	2.55	0.66	4.03	0.33	−1.44	2.29
	(0.00)	(0.11)	(0.00)	(0.15)	(0.00)	(0.07)	(0.06)
LWZ23	0.41	1.37	0.64	1.78	0.37	−0.96	1.40
	(0.00)	(0.30)	(0.00)	(0.32)	(0.00)	(0.18)	(0.16)
MWA23	0.42	2.44	0.66	2.60	0.34	−1.47	1.70
	(0.00)	(0.13)	(0.00)	(0.17)	(0.00)	(0.02)	(0.06)

## Data Availability

The original contributions presented in the study are included in the article; further inquiries can be directed to the corresponding author.
